# Regional cerebral oxygen saturation during initial mobilization of critically ill patients is associated with clinical outcomes: a prospective observational study

**DOI:** 10.1186/s40635-025-00722-2

**Published:** 2025-02-03

**Authors:** Ryota Imai, Takafumi Abe, Kentaro Iwata, Seigo Yamaguchi, Takeshi Kitai, Atsuhiro Tsubaki

**Affiliations:** 1 Department of Rehabilitation, Uonuma Kikan Hospital, Minamiuonuma, Japan; 2 Department of Rehabilitation, Kobe City Medical Center General Hospital, Kobe, Japan; 3 Department of Emergency and Critical Care, Uonuma Kikan Hospital, Minamiuonuma, Japan; 4 Department of Cardiovascular Medicine, National Cerebral and Cardiovascular Center, Suita, Japan; 5 Institute for Human Movement and Medical Sciences, Niigata University of Health and Welfare, 1398 Shimami-cho, Kita-ku, Niigata, 950-3198 Japan

**Keywords:** Critically ill patients, Early mobilization, Near-infrared spectroscopy, Regional cerebral oxygen saturation, Rehabilitation

## Abstract

**Background:**

Vital signs help determine the safety of early mobilization in critically ill patients in intensive care units. However, none of these variables directly assess cerebral circulation. Therefore, we aimed to investigate the relationship of regional cerebral oxygen saturation (rSO_2_) and vital signs with in-hospital death in critically ill patients.

**Methods:**

This prospective study included critically ill patients admitted to the Uonuma Kikan Hospital Emergency Center who received physical therapy between June 2020 and December 2022. We continuously measured rSO_2_ during the initial mobilization using a wearable brain near-infrared spectroscopy device. With in-hospital death as the primary endpoint, the association between rSO_2_ and in-hospital death was assessed in Analysis 1 to determine the rSO_2_ cut-off value that predicts in-hospital death. In Analysis 2, patients were categorised into survival and non-survival groups to examine the temporal changes in vital signs and rSO_2_ associated with postural changes during mobilization.

**Results:**

Of the 132 eligible patients, 98 were included in Analysis 1, and 70 were included in Analysis 2. Analysis 1 demonstrated that lower premobilization rSO_2_ was independently associated with in-hospital death (odds ratio 0.835, 95% confidence interval 0.724–0.961, *p* = 0.012). Receiver operating characteristic curve analysis identified an optimal rSO_2_ cut-off value of 57% for predicting in-hospital death (area under the curve 0.818, sensitivity 73%, specificity 83%). Analysis 2 showed that rSO_2_ changes during mobilization were unrelated to changes in vital signs, suggesting rSO_2_ as an independent prognostic marker.

**Conclusions:**

The results suggest that rSO_2_ measured during initial mobilization is associated with in-hospital death in critically ill patients.

**Supplementary Information:**

The online version contains supplementary material available at 10.1186/s40635-025-00722-2.

## Background

Critically ill patients in intensive care units (ICUs) frequently require complex interventions to manage their consciousness, respiratory functions, and circulatory dynamics. Ensuring the safety of these patients during rehabilitation and mobilization is essential, and monitoring their condition before, during, and after mobilization enables rapid detection of adverse changes [1].

Early rehabilitation, which is safe and well-tolerated, results in better functional outcomes at hospital discharge, a shorter duration of delirium, and more ventilator-free days than standard care for severely ill patients [2]. Although early rehabilitation greatly improves short-term physical outcomes, its effectiveness in preventing post-intensive care syndrome is limited. Early rehabilitation of severely ill patients reportedly has no significant effect on cognitive function, mental health-related outcomes, or mortality [3], probably because it is implemented when patients are judged capable of mobilization based on various indicators and numerical values reflecting their physical condition, as indicated in a report by the Japanese Society of Intensive Care Medicine, titled “Evidence based expert consensus for early rehabilitation in the intensive care unit” [4]. However, pathophysiologies that cannot be identified based on these indicators may also be present. Identifying additional indicators may further improve patient safety and prognostic evaluations.

Criteria for the termination or continuation of early mobilization and active exercising in severely ill patients have been described depending on their respiratory status, circulatory status, consciousness, and subjective symptoms [5–7]. However, brain function assessments are typically subjective, relying on consciousness scales and patient responses, with no direct objective indicator available. This study explores near-infrared spectroscopy (NIRS) as a potential tool for real-time brain monitoring during mobilization.

NIRS, a noninvasive technique that uses near-infrared light, measures cerebral oxygenation and has been widely utilised in cardiac surgery [8, 9]. This method provides continuous, bedside monitoring of regional cerebral oxygen saturation (rSO_2_) [10], and recent research in ICUs has shown that low rSO_2_ correlates with negative outcomes, including failed breathing trials and increased delirium incidence [11–13]. These findings highlight the potential value of rSO_2_ in assessing cerebral circulation during intensive care.

However, compared with vital signs, including blood pressure (BP), heart rate (HR), and percutaneous arterial oxygen saturation (SpO_2_), which are frequently used for routine assessments, rSO_2_ values during mobilization are rarely measured, and little is known about their validity to monitor the mobilization of critically ill patients. To the best of our knowledge, no studies have assessed the association between rSO_2_ and clinical outcomes of patients undergoing early mobilization. Thus, we hypothesised that rSO_2_, an indicator of cerebral circulation, might be strongly associated with clinical outcomes in patients who are early mobilized, independently of general vital signs.

Accordingly, this study’s primary objective was to examine whether premobilization rSO_2_ correlates with in-hospital death in critically ill patients. A secondary objective was to investigate the temporal changes in rSO_2_ and other vital signs during mobilization.

## Methods

### Participants

This prospective study included patients who were admitted to the Department of Emergency and Critical Care Center at Uonuma Kikan Hospital and who were prescribed physiotherapy between June 2020 and December 2022. In this study, critically ill patients were defined as those who stayed in the ICU for at least 48 h. The eligibility criteria were as follows: ability to start mobilization according to a doctor’s decision that was based on the criteria for early mobilization or initiation of active exercise from an early stage [4] and acquisition of consent for participation from the patients themselves or their family members. Patients were excluded if they spent < 48 h in ICU, if they were aged < 18 years, if their body mass index (BMI) exceeded 35 kg/m^2^, if they had new-onset cerebral strokes, if their rSO_2_ could not be measured because of a poorly fitting probe for NIRS, or if they refused active treatment.

Patients who met these eligibility criteria were selected and grouped into tertiles based on their rSO_2_ values, and the patient backgrounds were compared. Subsequently, the association between rSO_2_ before initial mobilization and in-hospital death was investigated (Analysis 1). To investigate the temporal changes in rSO_2_ and other vital signs before and during initial mobilization (Analysis 2), patients whose measurements were interrupted during mobilization were excluded. Missing data were primarily caused by interruptions in measurement due to the displacement of the NIRS probe during mobilization or by the premature termination of mobilization owing to clinical conditions such as hypotension. These patients were excluded because these interruptions made it impossible to accurately analyse the changes during mobilization.

### Variables

Basic patient data, including age, sex, BMI, Acute Physiology and Chronic Health Evaluation II (APACHE II) score on ICU admission, underlying diseases, and the cause of death for in-hospital fatalities, were collected from medical records. Data regarding previous ventilator use, oxygen administration, catecholamine use, number of days to initial mobilization, duration of ICU stay, and blood haemoglobin (Hb) concentration at the start of initial mobilization were also collected.

### Mobilization

**Fig. 1:**
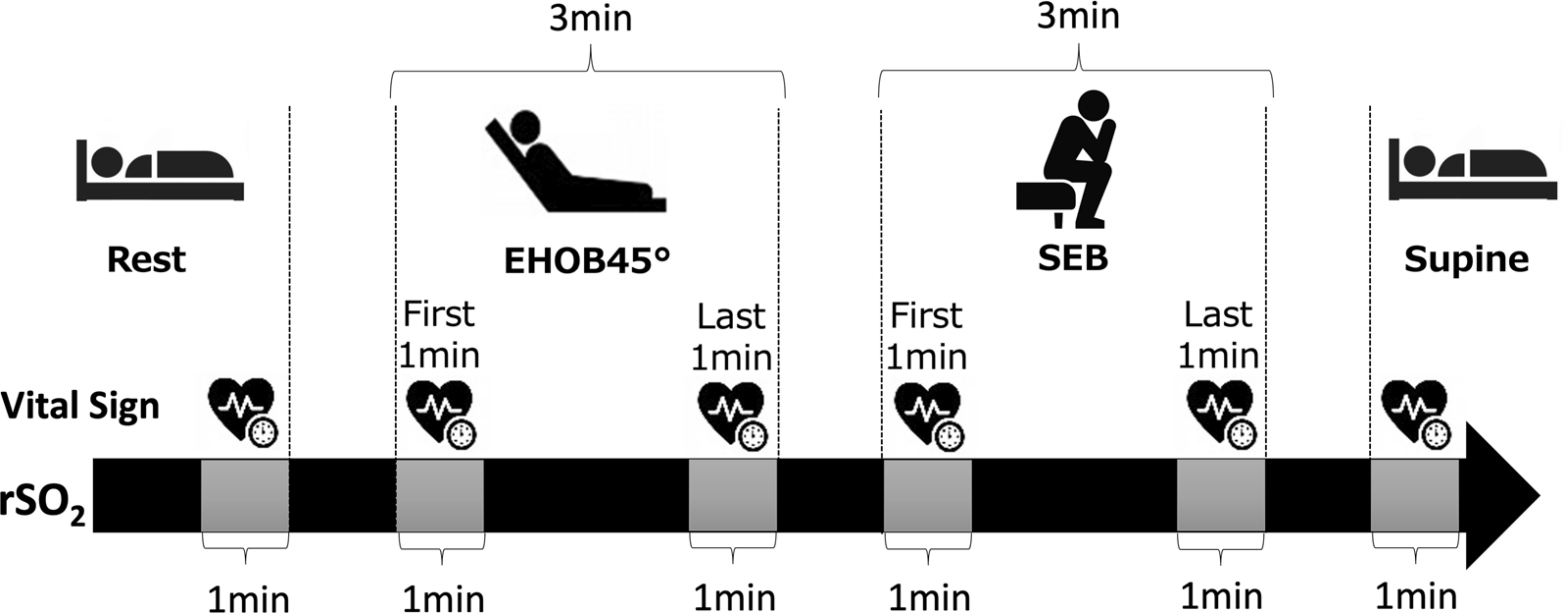
Mobilization procedure. The patient first lies down in the supine position and rests. The head end of the bed is then raised to 45°. Next, the patient sits on the edge of the bed. Each step lasts for 3 min. The rSO_2_ values are measured continuously from the resting position until mobilization is complete. Blood pressure and transcutaneous oxygen saturation are measured at six points: at rest, during the first 1 min of EHOB45°, during the last 1 min of EHOB45°, during the first 1 min of SEB, during the last 1 min of SEB, and while lying on one’s back. The rSO_2_ value is calculated as the average value over 1 min at the same six points. EHOB45°: head of the bed elevated to 45°; rSO_2_: regional cerebral oxygen saturation; SEB: sitting on the edge of the bed

In this study, mobilization was defined as rehabilitation at the level of sitting on the edge of the bed (SEB; sitting position) or more [14]. First, the patients lay supine on the bed to relax for 5 min; rSO_2_ measurement was initiated 1 min before the start of mobilization. The HR, BP, and SpO_2_ were also measured. Figure 1 shows the mobilization procedure. It involved resting (supine), laying with the head of the bed elevated to 45° (EHOB45°), and SEB. The duration of each step was 3 min. This duration was determined based on early mobilization protocols tailored for critically ill patients. Particularly, extended physical exertion poses risks in the ICU, and short-duration mobilization is recommended, according to the study by Schweickert et al. [2]. Additionally, the Critical Care Guidelines (2018) advise beginning mobilization with brief, manageable sessions to assess patient tolerance [15]. This approach enables patients to adopt a seated position safely and within their limits. The rSO_2_, HR, BP, and SpO_2_ were measured from the rest period before the start of mobilization until the patient was once again supine at the end of mobilization. Head and torso movements were minimised during the procedure.

### rSO_2_ measurements

The rSO_2_ was continuously measured using a wearable brain NIRS device (Brain Activity Monitor Hb133, Astem, Kanagawa, Japan) with two measurement channels having peak wavelengths of 770 ± 10 and 830 ± 10 nm, respectively. The distance between the probes was 35 mm. This device reportedly measures the status of oxygenation on the brain’s surface at a depth of approximately 12–20 mm beneath the skin [16]. The region of interest was the prefrontal cortex, which is not covered by hair and is considered to play a critical role in the ability to selectively allocate attention and integrate visual and proprioceptive information [17]. To ensure that the measurement sites were consistent, we fitted a special head mount to the patient’s forehead so that the probes were in close contact with the prefrontal cortex locations FP1 and FP2 according to the international 10–20 system [18], with measurements continuously obtained from start to finish of the initial mobilization. The sampling rate was 10 times per second (10 Hz), and the measured data were recorded on a computer via Bluetooth. The FP1 and FP2 measurement data were averaged and used as the rSO_2_ values before and after changing position for each of the following: rest before mobilization, during the first 1 min of EHOB45°, during the last 1 min of EHOB45°, during the first 1 min of SEB, and during the last 1 min of SEB, and during the 1 min in the supine position at the end of mobilization.

### Measurements of vital signs

The vital signs used for risk assessment and management during mobilization included HR, systolic BP (SBP), diastolic BP (DBP), mean arterial pressure (MAP), and SpO_2_. An IntelliVue X2 patient monitor (Philips, Amsterdam) was used to monitor the patients. HR was measured using the three-lead monitoring method (lead II) with the electrode applied to the chest, and the SBP, DBP, and MAP were measured using a noninvasive BP cuff placed on the upper arm. The SpO_2_ was measured using a disposable probe worn in a way that it enclosed the tissue of any of the fingers at a point where it was 6–18 mm thick. Each of these indicators was measured and recorded by the patient monitor at the following six time points during the initial mobilization: rest before mobilization, during the first 1 min of EHOB45°, during the last 1 min of EHOB45°, during the first 1 min of SEB, during the last 1 min of SEB, and during the 1 min in the supine position at the end of mobilization (Fig. 1).

### Statistical analyses

Analysis 1: This analysis aimed to examine whether premobilization rSO_2_ value and the primary endpoint of in-hospital death are associated. To compare the characteristics of patients categorised into the three study groups based on their rSO_2_ values, we used a one-way analysis of variance and the Kruskal–Wallis test or Fisher’s exact test. The normality of the data was evaluated using the Shapiro–Wilk test. The vital signs before mobilization were analysed in the same way.

The association between premobilization rSO_2_ and in-hospital death was analysed using logistic regression analysis adjusted for age and the APACHE II score, with in-hospital death (survival/non-survival) and rSO_2_ as the target and explanatory variables, respectively. Collinearity between the variables was evaluated using the variance inflation factor. Receiver operating characteristic (ROC) curve analysis was used to evaluate the optimum cut-off value of rSO_2_ for in-hospital death. The Youden index was used as the criterion for deciding the cut-off value. Next, to evaluate the ability of rSO_2_ to predict death, we conducted a logistic regression analysis using in-hospital death (survivor/non-survivor) as the dependent variable and the conventional vital signs (HR, MAP, and SpO_2_) plus rSO_2_ as the independent variables. In our study, the variables selected for the statistical model were based on vital signs frequently used for risk assessment in critically ill patients during mobilization in the ICU. These indicators are considered essential elements for assessing physiological stability in severely ill patients and are often associated with prognostic evaluation [19, 20]. Specifically, HR, MAP, and SpO₂ are widely used as predictors of clinical outcomes in critically ill patients, and they formed the basis for our variable selection. These variables are crucial for evaluating the immediate physiological state of patients in critical care and for predicting long-term treatment outcomes.

Analysis 2: This analysis aimed to investigate the temporal changes in rSO_2_ and other vital signs during exercise.

Patients with interrupted measurements during mobilization were excluded, and the remaining patients were divided into the survival and non-survival groups. Patients who survived until discharge or who were transferred to another hospital were classified as survivors, whereas those who died in the hospital were classified as non-survivors. Patient characteristics, vital signs, and rSO_2_ before mobilization were compared between the two groups using the t-test and Mann–Whitney U or Fisher’s exact test. To investigate whether the patient groups in Analysis 2 and Analysis 1 were similar or not, the characteristics of the patients excluded from the analysis were compared with those of the patients included in Analysis 2.

Two-way repeated measures analysis of variance was conducted to investigate changes over time in vital signs and rSO_2_ associated with changes in the position during mobilization. Additionally, correlations between changes in vital signs and those in rSO_2_ at each of the six measurement points during mobilization were investigated by calculating Pearson’s correlation coefficients or Spearman’s rank correlation coefficients.

Because of the exploratory nature of this study, the sample size was not calculated in advance. All statistical analyses were conducted using EZR [21] (EZR on R commander version 1.54), a graphical user interface of R (The R Foundation for Statistical Computing, Vienna, Austria). A *p*-value of < 0.05 was considered statistically significant in all analyses.

### Research ethics

The Institutional Review Board of Uonuma Kikan Hospital approved this study (approval number 02–006). Informed consent was obtained from all patients or their families. The study team at Uonuma Kikan Hospital conducted the study in accordance with the principles of the Declaration of Helsinki. Additionally, a record was created in the University Hospital Medical Information Network before data collection (record number R000040749).

## Results

**Fig. 2:**
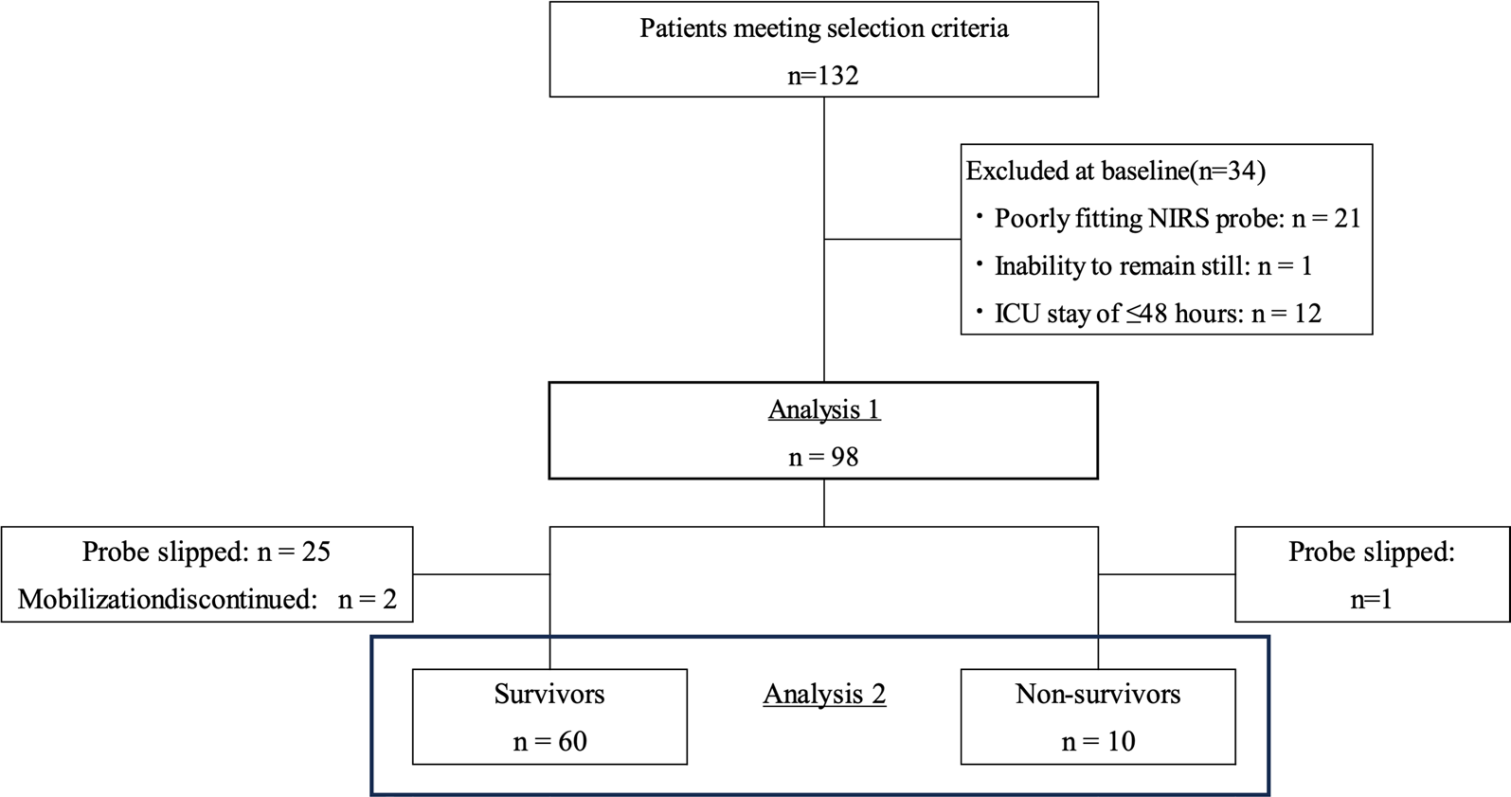
Patient enrolment flowchart. Between June 2020 and December 2022, 132 patients met the inclusion criteria. The head mount for NIRS did not fit well on 21 of these patients, and another patient could not remain still. Twelve more patients were excluded because they spent less than 48 h in the ICU. Finally, 98 patients were included in Analysis 1. In Analysis 2, 25 survivors were excluded because the probe slipped during mobilization, and two survivors were excluded because their mobilization was discontinued for reasons such as hypotension. One non-survivor was excluded because the probe slipped during mobilization. Thus, Analysis 2 included 60 survivors and 10 non-survivors. *ICU* intensive care unit, *NIRS* near-infrared spectroscopy

Figure 2 shows the patient enrolment flowchart. Between June 2020 and December 2022, 132 patients met the inclusion criteria. Of these patients, 21 (15.9%) for whom the NIRS head mount did not fit well and one (0.8%) who could not remain still were excluded. Another 12 (9.1%) patients were excluded because they spent < 48 h in the ICU. Finally, 98 patients were included in Analysis 1. In Analysis 2, 25 survivors were excluded because the probe slipped during mobilization, and two were excluded because their mobilization was discontinued for reasons such as hypotension. One non-survivor was excluded because the probe slipped during mobilization. Thus, Analysis 2 comprised 60 survivors and 10 non-survivors.

A comparison of the patients included in Analysis 2 and those excluded from it showed that the characteristics and vital signs of these two groups did not significantly differ apart from sex and Hb (Additional file 1:Supplementary Table 1). Moreover, the patient group included in Analysis 2 was equivalent to that in Analysis 1, and no significant differences were observed in demographic characteristics or clinical indicators in the dataset (Additional file 1:Supplementary Table 2).

### Results of analysis 1: association between premobilization rSO_2_ and in-hospital death

Table 1 presents the characteristics of the patients in this study. The overall mean age was 77 ± 11 years. The median time to first bed release was 3 days (interquartile range [IQR], 2–5), and the median length of hospital stay was 23 days (IQR, 13–40). No significant differences were found in age, sex, severity of illness, or BMI among the three groups classified based on rSO_2_. Hb levels were significantly higher in the High (10.1 ± 1.6 g/dL vs 11.7 ± 1.7 g/dL; *p* < 0.05) and Medium (10.1 ± 1.6 g/dL vs 10.1 ± 1.6 g/dL; *p* < 0.05) rSO_2_ groups than in the Low rSO_2_ group. The median number of days to initial mobilization was significantly shorter in the Medium rSO_2_ group (4 days [IQR, 3–6] vs 3 days [IQR, 2–5]; *p* < 0.05) than in the Low rSO_2_ group. No significant difference was observed between the two groups regarding oxygenation during transfers; however, the number of in-hospital deaths was significantly higher in the Low rSO_2_ group than in the High (8 vs 0 patients; *p* < 0.05) and Medium (8 vs 3 patients; *p* < 0.05) rSO_2_ groups. However, the three study groups did not significantly differ in the vital signs measured before mobilization (Table 2).

**Table 1:** Characteristics of patients

	Total (n = 98)	Low rSO_2_ group	Medium rSO_2_ group	High rSO_2_ group	*p*-value
		< 57%	57% ≤ rSO_2_ ≤ 62%	> 62%	
		(n = 25)	(n = 46)	(n = 27)	
Age, year^a^	77 ± 11	76 ± 13	77 ± 11	77 ± 10	0.995
Sex, female, n (%)^b^	36 (37%)	9 (36%)	17 (37%)	10 (37%)	> 0.999
APACHE II score^a^	19.1 ± 6.9	21.2 ± 7.6	18.3 ± 7.4	18.4 ± 5.2	0.213
BMI (kg/m^2^)^a^	22.8 ± 3.6	22.7 ± 3.4	22.7 ± 3.8	23.1 ± 3.6	0.891
Hb (g/dL)^a^	10.7 ± 1.7	10.1 ± 1.6†	10.5 ± 1.6‡	11.7 ± 1.7	0.003
Time to initial mobilization (days)^c^	3 (2–5)	4 (3–6)*	3 (2–5)	3 (2–6)	0.028
ICU stay (days)^c^	6 (4–9)	7 (6–11)	6 (4–9)	5 (4–10)	0.087
Hospital stay (days)^c^	23 (13–40)	24 (17–48)	21 (12–35)	22 (13–44)	0.586
Ventilator use, n (%)^b^	44 (45%)	12 (48%)	19 (41%)	13 (48%)	0.78
Catecholamine use, n (%)^b^	53 (54%)	15 (60%)	24 (52%)	14 (52%)	0.838
Oxygenadministration, n (%)^b^	57 (58%)	14 (56%)	25 (54%)	16 (59%)	0.965
In-hospital death, n (%)^b^	11 (11%)	8 (32%)†*	3 (7%)	0 (0%)	< 0.001
Reason for ICU admission, n (%)					
Circulatory disease^b^	15 (15%)	5 (20%)	7 (15%)	3 (11%)	
Respiratory disease^b^	8 (8%)	3 (12%)	4 (9%)	1 (4%)	
Abdominal/gastrointestinal disease^b^	17 (17%)	4 (16%)	9 (20%)	4 (15%)	
Sepsis^b^	31 (32%)	10 (40%)	12 (26%)	9 (33%)	
Renal/metabolic disease^b^	13 (13%)	3 (12%)	6 (13%)	4 (15%)	
Trauma^b^	9 (9%)	0 (0%)	6 (13%)	3 (11%)	
Other^b^	5 (5%)	0 (0%)	2 (4%)	3 (11%)	

*APACHE II* Acute Physiology and Chronic Health Disease Classification System II, *BMI* body mass index, *Hb* haemoglobin, *ICU* intensive care unit, *rSO*_*2*_ regional cerebral oxygen saturation

^a^Values are shown as the mean ± SD

^b^Values are shown as the number of patients (%)

^c^Values are shown as the median (interquartile range)

^*^p < 0.05, Low rSO_2_ group vs Medium rSO_2_ group

^†^*p* < 0.05, Low rSO_2_ group vs High rSO_2_ group

^‡^*p* < 0.05, Medium rSO_2_ group vs High rSO_2_ group

**Table 2:** Comparison of vital signs before mobilization

	Total (n = 98)	Low rSO_2_ group	Medium rSO_2_ group	High rSO_2_ group	*p*-value
		< 57%	57% ≤ rSO_2_ ≤ 62%	> 62%	
		(n = 25)	(n = 46)	(n = 27)	
HR (bpm)	83 ± 17	87 ± 19	79 ± 16	87 ± 16	0.050
SBP (mmHg)	127 ± 25	126 ± 28	124 ± 24	134 ± 25	0.197
DBP (mmHg)	65 ± 14	69 ± 13	62 ± 13	68 ± 15	0.075
MAP (mmHg)	85 ± 16	86 ± 17	82 ± 15	89 ± 17	0.143
SpO_2_ (%)	96 ± 2	96 ± 2	96 ± 2	97 ± 2	0.334

Values are shown as the mean ± SD

*DBP* diastolic blood pressure, *HR* heart rate, *MAP* mean arterial pressure, *rSO*_*2*_ regional cerebral oxygen saturation, *SBP* systolic blood pressure, *SpO*_*2*_ percutaneous arterial oxygen saturation

**Fig. 3:**
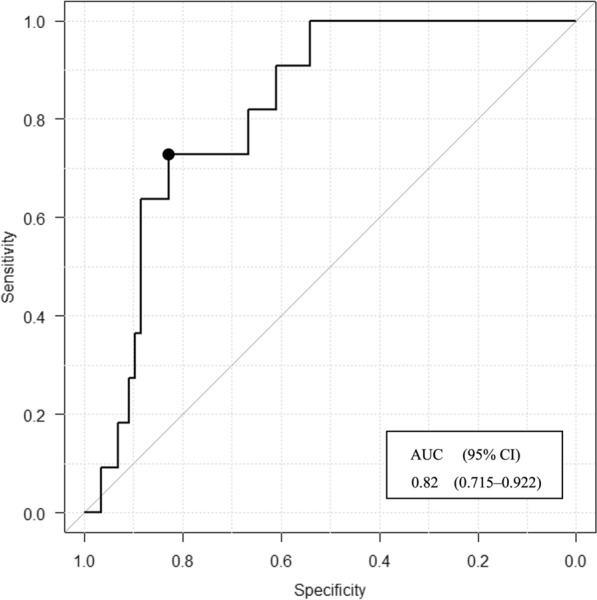
Receiver operating characteristic curve analysis for in-hospital death. The rSO_2_ cut-off value for predicting in-hospital death is 57% (specificity, 0.83; sensitivity, 0.73; AUC, 0.82; 95% CI 0.715–0.922). The filled circle on the curve indicates the cut-off value. *AUC* area under the curve, *CI* confidence interval, *rSO*_*2*_ regional cerebral oxygen saturation

Table 3 shows the results of the multiple logistic regression analysis with in-hospital death as the target variable. Variables were not multicollinear. Multiple logistic regression analysis showed that premobilization rSO_2_ was significantly associated with in-hospital death (odds ratio [OR], 0.835; 95% confidence interval [CI] 0.724–0.961; *p* = 0.012). This result suggested premobilization rSO_2_ as an independent predictor of in-hospital death. Figure 3 shows the ROC curve for rSO_2_ as a predictor of in-hospital death. The area under the curve (AUC) for rSO_2_ was 0.818 (95% CI 0.715–0.922), and the optimal cut-off value was 57% (specificity, 0.83; sensitivity, 0.73; positive predictive value, 0.35; negative predictive value, 0.96; positive likelihood ratio, 4.28; and negative likelihood ratio, 0.33).

**Table 3:** Logistic regression analysis with in-hospital death as the target variable

	Odds ratio	95% CI	*p*-value	VIF
Age	1.010	0.952–1.080	0.683	1.034
APACHE II	1.040	0.953–1.140	0.361	1.034
rSO_2_	0.835	0.724–0.961	0.012	1.001

Hosmer–Lemeshow test: chi-squared = 14.5236

363 (df = 8), *p* = 0.069

The Hosmer–Lemeshow test is used to evaluate the calibration of a model, and it evaluates a different aspect than indices such as discrimination and positive predictive value

Survivor/non-survivor grouping was the dependent variable

*APACHE II* Acute Physiology and Chronic Health Disease Classification System II, *CI* confidence interval, *rSO*_*2*_ regional cerebral oxygen saturation, *VIF* variance inflation factor

Table 4 presents the results of logistic regression analysis of conventional vital signs and rSO_2_. Pre-hospital rSO_2_ was significantly correlated with in-hospital mortality (OR, 0.836; 95% CI 0.725–0.964; *p* = 0.014), with an AUC of 0.822 (95% CI 0.727–0.918).

**Table 4:** Logistic regression analysis using conventional vital signs and rSO_2_

	Odds ratio	95% CI	*p*-value	VIF
HR	1.010	0.968–1.04	0.789	1.114
MAP	0.977	0.933–1.02	0.340	1.034
SpO_2_	1.100	0.751–1.60	0.634	1.145
rSO_2_	0.836	0.725–0.964	0.014	1.028

Area under the curve, 0.822; 95% CI, 0.727–0.918

Survivor/non-survivor grouping was the dependent variable

*CI* confidence interval, *HR* heart rate, *MAP* mean arterial pressure, *rSO*_*2*_ regional cerebral oxygen saturation, *SpO*_*2*_ percutaneous arterial oxygen saturation, *VIF*, variance inflation factor

### Results of analysis 2: association between changes in rSO_2_ and vital signs during mobilization

Table 5 shows the characteristics of the patients in Analysis 2. The median ICU stay was significantly longer for non-survivors (10 [IQR, 8–19] days) than for survivors (6 [IQR, 4–8] days; *p* = 0.002). No other item was significantly different between the two study groups. A comparison of vital signs and premobilization rSO_2_ revealed a significantly lower rSO_2_ in non-survivors than in survivors (*p* = 0.009; Table 6).

**Table 5:** Characteristics of patients in Analysis 2

	Total (n = 70)	Survivors (n = 60)	Non-survivors (n = 10)	*p-*value
Age, years^a^	76 ± 12	76 ± 12	77 ± 11	0.844
Sex, female, n (%)^b^	19 (27%)	17 (28%)	2 (20%)	0.717
APACHE II score^a^	18.7 ± 6.6	18.2 ± 6.4	21.4 ± 7.9	0.162
BMI (kg/m^2^)^a^	22.8 ± 3.4	22.9 ± 3.4	22.4 ± 3.6	0.406
Hb (g/dL)^a^	11.0 ± 1.7	11.1 ± 1.7	10.5 ± 1.8	0.335
Time to initial mobilization (days)^c^	3 (2–5)	3 (2–5)	4 (3–6)	0.243
ICU stay (days)^c^	6 (4–9)	6 (4–8)	10 (8–19)	0.002
Hospital stay (days)^c^	19 (12–40)	18 (12–36)	23 (13–72)	0.486
History of ventilator use, n (%)b	30 (43%)	27 (45%)	3 (30%)	0.498
Catecholamine use, n (%)^b^	34 (49%)	27 (45%)	7 (70%)	0.182
Oxygenadministration, n (%)^b^	42 (60%)	35 (58.3%)	7 (70%)	0.729
Reason for ICUadmission, n (%)				
Circulatory disease^b^	14 (20%)	12 (20%)	2 (20%)	
Respiratory disease^b^	7 (10%)	6 (10%)	1 (10%)	
Abdominal/gastrointestinal disease^b^	11 (16%)	7 (12%)	4 (40%)	
Sepsis^b^	17 (24%)	15 (25%)	2 (20%)	
Renal/metabolic disease^b^	11 (16%)	10 (17%)	1 (10%)	
Trauma^b^	6 (9%)	6 (10%)	0 (0%)	
Other^b^	4 (6%)	4 (7%)	0 (0%)	
Cause of death, n (%)				
Ventricular fibrillation^b^			1 (10%)	
Heart failure^b^			3 (30%)	
Pneumonia^b^			2 (20%)	
Liver failure^b^			1 (10%)	
Pancreatitis^b^			1 (10%)	
Cancer^b^			1 (10%)	
Sepsis^b^			1 (10%)	

*APACHE II* Acute Physiology and Chronic Health Disease Classification System II, *BMI* body mass index, *Hb* haemoglobin, *ICU* intensive care unit

^a^Values are shown as the mean ± SD

^b^Values are shown as the number of patients (%)

^c^Values are shown as the median (interquartile range)

**Table 6:** Comparison of premobilization vital signs and rSO_2_ between groups in Analysis 2

	Survivors (n = 70)	Non-survivors (n = 10)	*p-*value
HR	84 ± 15	85 ± 25	0.799
SBP	130 ± 25	114 ± 26	0.079
DBP	66 ± 14	61 ± 6	0.330
MAP	87 ± 16	77 ± 10	0.078
SpO_2_	96 ± 2	97 ± 2	0.789
rSO_2_	60 ± 5	56 ± 3	0.009

Values are shown as the mean ± SD.

*DBP* diastolic blood pressure, *HR* heart rate, *MAP* mean arterial pressure, *rSO*_*2*_ regional cerebral oxygen saturation, *SBP* systolic blood pressure, *SpO*_*2*_ percutaneous arterial oxygen saturation

**Fig. 4:**
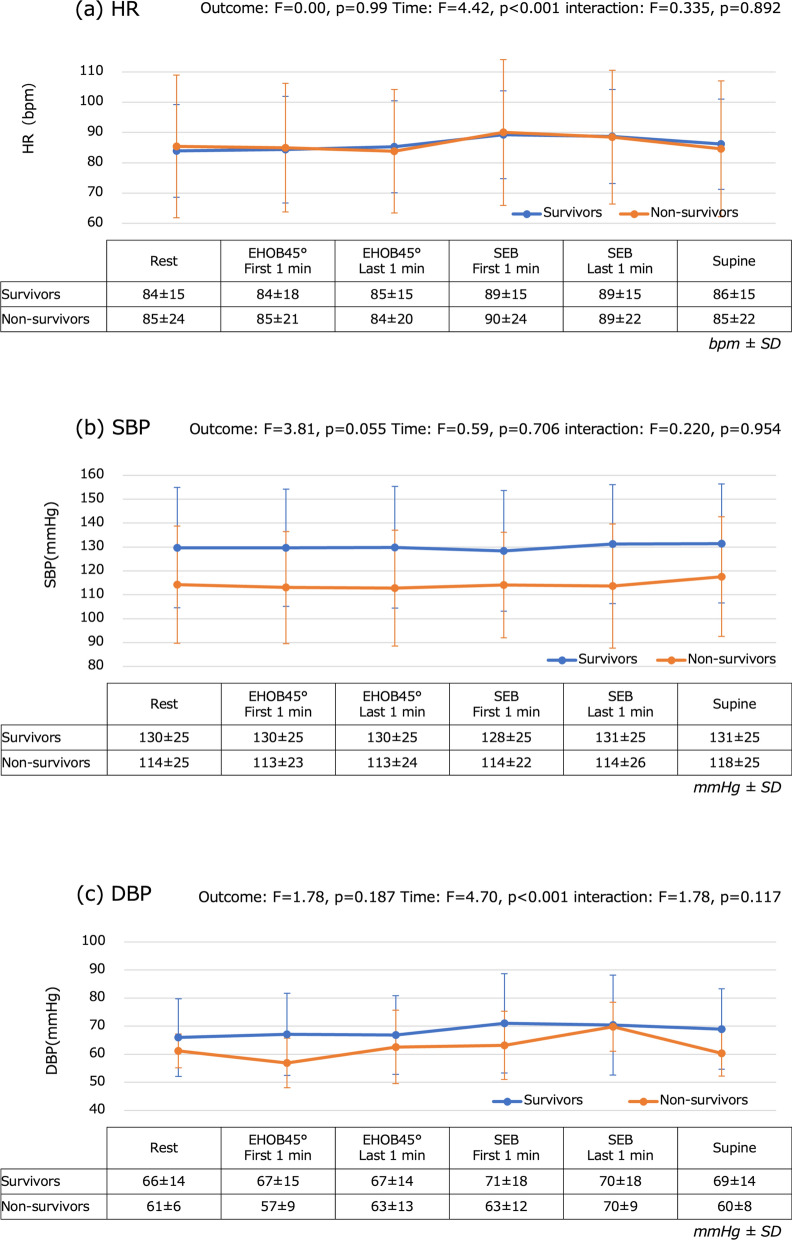

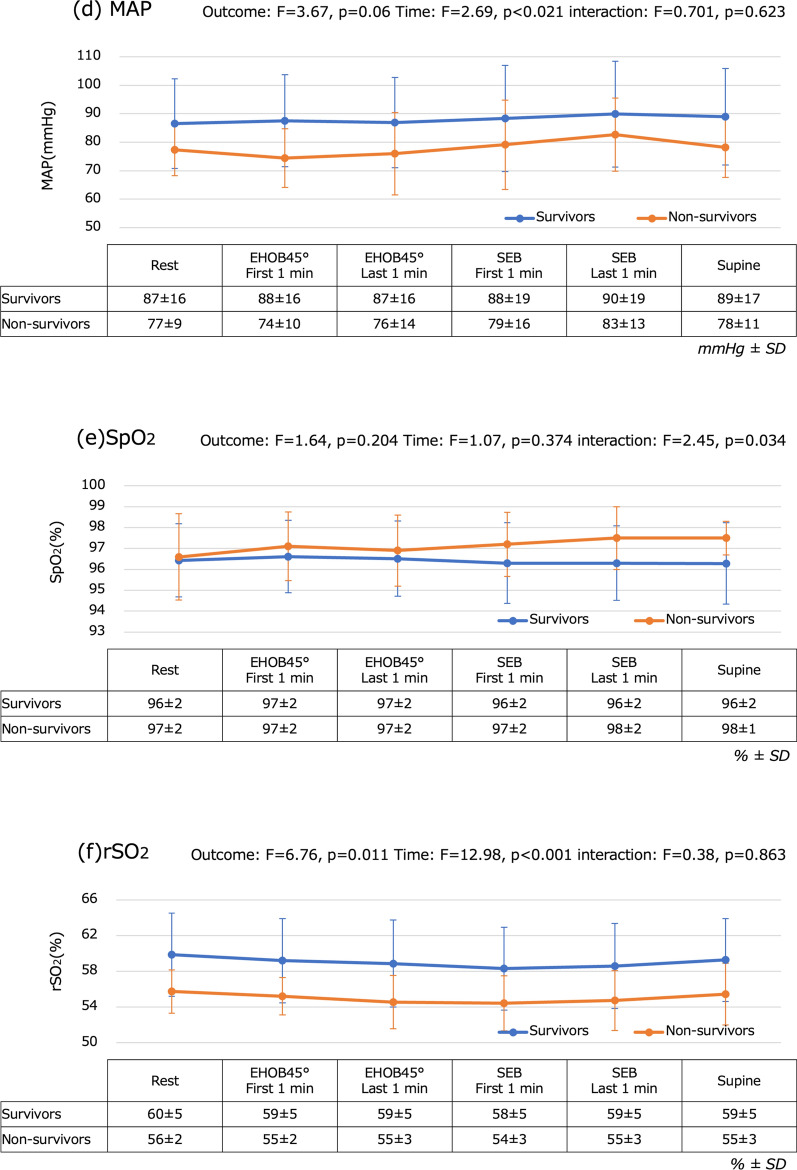
Temporal changes in vital signs and regional cerebral oxygen saturation. **a** HR is highest at SEB first minute (survivors: 89 ± 14 bpm, non-survivors: 90 ± 24 bpm). This change is significant (*p* < 0.001). **b** SBP does not change significantly with position. **c** DBP is highest during the first minute of SEB (survivors, 71 ± 18 mmHg; non-survivors, 70 ± 9 mmHg). This change is significant (*p* < 0.001). **d** MAP is highest during the last minute of SEB (survivors, 90 ± 19 mmHg; non-survivors, 83 ± 13 mmHg). This change is significant (*p* = 0.021). **e** SpO_2_ does not change significantly with position. **f** rSO_2_ is lowest at SEB first minute (survivors, 58% ± 5%; non-survivors, 54 ± 3 mmHg). This change is significant (*p* < 0.001). The rSO2 values of the two study groups were also significantly different at this time point (*p* = 0.011). DBP: diastolic blood pressure; EHOB45°: head of the bed elevated to 45°; HR: heart rate; MAP: mean arterial pressure; rSO_2_: regional cerebral oxygen saturation; SBP: systolic blood pressure; SEB: sitting on the edge of the bed; SpO_2_: transcutaneous arterial oxygen saturation

Figure 4 shows the changes over time in vital signs and rSO_2_ that were associated with changes in position during mobilization. SBP and SpO_2_ did not significantly change. HR was the highest during the first 1 min of SEB (survivors, 89 ± 14 bpm; non-survivors, 90 ± 24 bpm). DBP was the highest during the first 1 min of SEB (survivors, 71 ± 18 mmHg; non-survivors, 70 ± 9 mmHg), whereas MAP was the highest during the last 1 min of SEB (survivors, 88 ± 19 mmHg; non-survivors, 79 ± 16 mmHg). The changes in HR, DBP, and MAP were all significant (*p* < 0.001 for HR and DBP, *p* = 0.021 for MAP). However, no significant differences were found between survivors and non-survivors in any of these cases. The rSO_2_ was the lowest during the first 1 min of SEB (survivors, 58 ± 5%; non-survivors, 54 ± 3%), a significant change (*p* < 0.001), and the value was significantly lower for non-survivors than for survivors (*p* = 0.011). Next, we evaluated the relationship between changes in vital signs and those in rSO_2_ at each measurement point in the mobilization process. We performed correlation analyses at multiple measurement points; however, after applying Bonferroni correction, we found no strong relationship between changes in rSO_2_ and those in vital signs (Additional file 1:Supplementary Tables 3 and 4).

## Discussion

In this study, an exploratory analysis of patient groups categorised into tertiles of rSO₂ revealed an association between low premobilization rSO₂ levels and in-hospital death in critically ill patients. First, premobilization rSO_2_ was independently associated with the outcome of in-hospital death, with a cut-off value of 57% for predicting in-hospital death. Second, no strong correlation was observed between changes in rSO_2_ and those in conventional vital signs, and the changes varied depending on the time points.

In a previous study, the rSO_2_ in patients in a state of shock at 72 h after admission was significantly lower in non-survivors than in survivors [22]. Another study in patients with septic shock showed that the minimum rSO_2_ was significantly lower in non-survivors than in survivors [23]. A systematic review of NIRS measurement during cardiac arrest also showed that the recovery of spontaneous circulation is associated with rSO_2_ and that patients with higher rSO_2_ during resuscitation survived to discharge and exhibited good neurological outcomes [24]. Similarly, in this study, we found that rSO_2_ was lower in non-survivors, and the evidence from previous studies supports our results. Additionally, the differences in premobilization rSO₂ values may be associated with the preservation of cerebral autoregulation functions between survivors and non-survivors. In patients with intact autoregulation, cerebral blood flow remains stable within a certain range, ensuring a consistent supply of oxygen and nutrients to brain tissues. In contrast, in critically ill patients, especially non-survivors, this autoregulatory function is frequently compromised [25], leading to unstable cerebral blood flow and a tendency toward lower local oxygen saturation. This dysfunction can be further exacerbated by systemic inflammatory responses and fluctuations in BP [26]. Furthermore, disruptions in neurovascular coupling may affect rSO₂ values. This process, which typically increases blood flow in response to heightened neural activity, may be insufficient in patients with impaired neurological functions [27]. Consequently, non-survivors may lack the appropriate increase in blood flow that corresponds to neural activity, maintaining relatively lower rSO_2_ values. These physiological differences offer critical insights for adapting patient management and therapeutic strategies, particularly within intensive care settings. Oxygen administration is commonly used to manage hypoxemia in critically ill patients; however, it can impact rSO_2_ values measured using NIRS. While supplemental oxygen improves SpO_2_, it may not accurately reflect cerebral oxygenation, especially in patients with impaired cerebral autoregulation. In non-survivors, this autoregulatory function is frequently compromised, potentially causing a discrepancy between systemic oxygen levels and cerebral oxygenation [28]. NIRS measures rSO_2_ based on the near-infrared light absorption of oxyhaemoglobin and deoxyhaemoglobin in brain tissue. Artificial increases in systemic oxygenation can sometimes mask actual cerebral hypoxia. This discrepancy, particularly in non-survivors with reduced cerebral blood flow despite high peripheral oxygen saturation, can lead to an underestimation of cerebral hypoxia severity [29]. Therefore, the improved SpO₂ values resulting from oxygen supplementation do not necessarily indicate improved rSO₂. It is crucial in clinical evaluations to interpret rSO_2_ values within the broader context of the patient’s overall physiological state.

In our study population, the optimum rSO_2_ cut-off value for predicting in-hospital death was 57% (AUC, 0.82; sensitivity, 0.73; specificity, 0.83), and the predictive power of rSO_2_ was significantly better than that of conventional vital signs. In previous studies, the reported cut-off value of baseline rSO_2_ for in-hospital death among patients who had undergone major cardiovascular surgery was 50.5% (AUC, 0.715; sensitivity, 50.0%; specificity, 92.2%) [30], the reported optimum NIRS-based cut-off value for predicting a good outcome after subarachnoid haemorrhage was 63% (AUC, 0.86; sensitivity, 1.00; specificity, 0.63) [31], and the reported optimum cut-off value for predicting good neurological outcomes at 90 days after out-of-hospital cardiac arrest was 42% (AUC, 0.90; sensitivity, 0.79; specificity, 0.95) [32], with the value generally remaining ≤ 60%. However, illness and measurement timing may contribute to differences in rSO_2_ cut-off values. Previous studies have also highlighted that the absolute rSO_2_ value varies between different measurement devices [33–35], probably because of factors such as different measurement methods and distances between probes. Thus, the results obtained with different devices should be interpreted with caution. In this study, the Youden index was used as the basis for selecting the cut-off value. This method is widely used as a standard for maximising the balance between sensitivity and specificity on the ROC curve, and the cut-off is selected as the point where the value obtained by subtracting 1 from the total sensitivity and specificity is maximised. This approach makes it possible to set a cut-off that considers both sensitivity and specificity. We recognise that in clinical decision-making in the ICU, it is sometimes important to prioritise sensitivity and avoid false negatives. However, if the cut-off is set relatively low, there is a concern that most patients may be judged to be at risk, reducing its practicality in clinical settings. In this study, we adopted the Youden index, which considers the balance between sensitivity and specificity, and focused on setting a cut-off that is useful in clinical settings. In future research, we will also consider different cut-off selection criteria according to specific clinical situations in the ICU, and if an approach that prioritises sensitivity is required, we will analyse it to minimise the risk of false negatives. Additionally, this study did not evaluate the appropriateness of the cut-off value. To evaluate the appropriateness of the rSO_2_ cut-off value, further external validation in another independent population is needed, and it is important to assess the generalisability of this cut-off value by validating it in other populations in future studies.

Although a significant difference was observed in the change in vital signs measured using rSO_2_ and the conventional method at only some points, after applying Bonferroni correction, no statistically significant association was found between the change in rSO_2_ and that in vital signs. This result suggests that the change in rSO_2_ occurs independently of that in SpO_2_ or MAP. A study of the cerebral oxygenation response to postural changes in healthy individuals found that the MAP and rSO_2_ exhibited similar patterns of changes, whereas the SpO_2_ did not significantly change compared with its baseline value [36]. However, a previous study of seriously ill patients in a state of shock or requiring mechanical ventilation found no association between MAP and rSO_2_ [37]. A study of patients who underwent thoracic surgery under single-lung ventilation and received interventions to maintain SpO_2_ at ≥ 90% found that SpO_2_ was not significantly correlated with rSO_2_ [38]. These previous results and the findings of this study collectively indicate that rSO_2_ may not be associated with SpO_2_, irrespective of the disease and any interventions, and that rSO_2_ may vary independently of MAP, even in patients with serious conditions and those who undergo interventions to maintain organ perfusion. Additionally, the fact that the peaks of DBP and MAP occurred at different times in the change over time of the vital signs, with no change in SBP, may be due to random fluctuations, making it difficult to conclude that there was a significant physiological change. Further investigation is needed to determine whether the fluctuations were due to chance factors or not.

This study excluded patients with missing rSO_2_ data during mobilization. Missing data primarily arose from probe displacement or technical issues inherent to ICU settings. The exclusion of patients was necessary to ensure the robustness of the dataset and the accuracy of the results, as imputing values for physiological measurements might introduce assumptions that may compromise the validity of the findings. We acknowledge that missing data can affect the generalizability of the results. However, consistent with prior studies investigating early mobilization in critically ill patients [2], we opted for a complete-case analysis to focus on directly observed relationships between rSO_2_ and clinical outcomes. This approach aligns with the study’s primary objective of exploring rSO_2_ as a reliable, real-time indicator during mobilization. A comparative analysis showed no significant differences in most characteristics between included and excluded patients, suggesting a minimal effect on generalizability.

This study had some limitations. First, the study population included only 11 non-survivors. However, this is an exploratory study, and these results should be verified in detail, including survival curve analysis, in a properly designed large-scale study. Second, the study participants were ICU-admitted patients with severe illnesses from various backgrounds; therefore, their conditions were not uniform. Third, vital signs were only measured at six time points; thus, transient changes and drops may not have shown up in the results. Fourth, given the specific nature of rehabilitation in a clinical setting, it was impossible not to talk to or address the patients, and their movements could not be fully controlled. Fifth, the NIRS signal is greatly affected by blood flow in the extracranial skin [39], and this effect could not be excluded in this study. Sixth, patients with missing rSO₂ data during mobilization were excluded from Analysis 2 due to the inability to assess changes accurately. While this ensured the validity of the analysis, it may have introduced potential selection bias. Further studies in patients stratified based on disease or condition and multicentre prospective cohort studies will be required to ascertain whether the measurement of oxygenation dynamics by NIRS facilitates the assessment of critically ill patients during early mobilization. Additionally, the data collection methods should be improved to minimize data loss during mobilization. Nevertheless, this study identified rSO_2_ at the start of mobilization as a new indicator associated with patient outcome and determined the rSO_2_ cut-off value; therefore, the findings are clinically meaningful.

## Conclusions

The results of this study indicate that the rSO_2_ value measured using NIRS during initial mobilization is associated with in-hospital death in critically ill patients. This finding suggests that rSO_2_, as a noninvasive measurement, could provide valuable insights into patient outcomes in the ICU setting. Further studies are warranted to confirm these associations and explore the clinical applicability of rSO_2_ in early mobilization strategies for critically ill patients.

## Supplementary Information

Additional file 1

## Data Availability

The datasets generated during and/or analysed during the current study are not publicly available because they may be used for retrospective analysis by the co-authors but are available from the corresponding author upon reasonable request.

## Abbreviations

APACHE II: Acute Physiology and Chronic Health Evaluation II
AUC: Area under the curve
BMI: Body mass index
BP: Blood pressure
CI: Confidence interval
DBP: Diastolic blood pressure
EHOB45°: Head of the bed elevated to 45°
Hb: Haemoglobin
HR: Heart rate
ICU: Intensive care unit
IQR: Interquartile range
MAP: Mean arterial pressure
NIRS: Near-infrared spectroscopy
OR: Odds ratio
ROC: Receiver operating characteristic
rSO_2_: Regional cerebral oxygen saturation
SBP: Systolic blood pressure
SEB: Sitting on the edge of the bed
SpO_2_: Percutaneous arterial oxygen saturation
