# Supplementary material for: Regional cerebral oxygen saturation during initial mobilization of critically ill patients is associated with clinical outcomes: a prospective observational study
Source: Intensive Care Med Exp. 2025 Feb 3;13:13. doi: 10.1186/s40635-025-00722-2 (PMC11790542; doi:10.1186/s40635-025-00722-2)
Supplement: Supplementary file 1 — Additional file 1 [file 40635_2025_722_MOESM1_ESM.docx]

**Supplementary Table 1** Comparison of the characteristics of patients included in Analysis 2 and those excluded from it

|  | Included patients | Excluded patients | *p*-value |
| --- | --- | --- | --- |
|  | (n = 70) | (n = 28) |  |
| Age, year ^a^ | 76 ± 12 | 78 ± 10 | 0.729 |
| Sex, female, n (%) ^b^ | 19 (27%) | 17 (61%) | 0.003 |
| APACHE II score ^a^ | 18.7 ± 6.6 | 20.1 ± 7.7 | 0.576 |
| BMI (kg/m^2^) ^a^ | 22.8 ± 3.4 | 22.8 ± 4.0 | 0.943 |
| Hb (g/dL) ^a^ | 11.0 ± 1.7 | 10.0 ± 1.5 | 0.005 |
| Time to initial mobilization (days) ^c^ | 3 (2–5) | 3 (2–5) | 0.652 |
| ICU stay (days) ^c^ | 6 (4–9) | 6 (4–10) | 0.921 |
| Hospital stay (days) ^c^ | 19 (12–40) | 29 (18–40) | 0.092 |
| Ventilator use, n (%) ^b^ | 30 (43%) | 14 (50%) | 0.654 |
| Catecholamine use, n (%) ^b^ | 34 (49%) | 19 (68%) | 0.116 |
| Oxygen administration, n (%) ^b^ | 40 (57%) | 14 (50%) | 0.654 |
| In-hospital death, n (%) ^b^ | 10 (14%) | 1 (4%) | 0.171 |
| Premobilization vital signs |  |  |  |
| rSO_2_ (%) ^a^ | 59 ± 5 | 60 ± 4 | 0.847 |
| HR (bpm) ^a^ | 84 ± 17 | 80 ± 18 | 0.345 |
| SBP (mmHg) ^a^ | 128 ± 26 | 127 ± 25 | 0.886 |
| DBP (mmHg) ^a^ | 65 ± 13 | 65 ± 16 | 0.953 |
| MAP (mmHg) ^a^ | 85 ± 15 | 84 ± 18 | 0.780 |
| SpO_2_ (%) ^a^ | 96 ± 2 | 96 ± 2 | 0.761 |

^a^Values are shown as the mean ± SD.

^b^Values are shown as the number of patients (%).

^c^Values are shown as the median (interquartile range).

APACHE II: Acute Physiology and Chronic Health Disease Classification System II; BMI: body mass index; DBP: diastolic blood pressure; Hb: haemoglobin; HR: heart rate; ICU: intensive care unit; MAP: mean arterial pressure; rSO_2_: regional cerebral oxygen saturation; SBP: systolic blood pressure; SpO_2_: percutaneous arterial oxygen saturation

**Supplementary Table 2** Comparison of the datasets of patient characteristics between Analysis 1 and Analysis 2

|  | Analysis 1 dataset | Analysis 2 dataset | *p*-value |  |
| --- | --- | --- | --- | --- |
|  |  |  |  |  |
|  | (n = 98) | (n = 70) |  |  |
| Age, year ^a^ | 77 ± 11 | 76 ± 12 | 0.887 |  |
| Sex, female, n (%) ^b^ | 36 (37%) | 19 (27%) | 0.243 |  |
| APACHE II score ^a^ | 19.1 ± 6.9 | 18.7 ± 6.6 | 0.819 |  |
| BMI (kg/m^2^) ^a^ | 22.8 ± 3.6 | 22.8 ± 3.4 | 0.976 |  |
| Hb (g/dL) ^a^ | 10.7 ± 1.7 | 11.0 ± 1.7 | 0.255 |  |
| Time to initial mobilization (days) ^c^ | 3 (2–5) | 3 (2–5) | 0.854 |  |
| ICU stay (days) ^c^ | 6 (4–9) | 6 (4–9) | 0.968 |  |
| Hospital stay (days) ^c^ | 23 (13–40) | 19 (12–40) | 0.490 |  |
| Ventilator use, n (%) ^b^ | 44 (45%) | 30 (43%) | 0.875 |  |
| Catecholamine use, n (%) ^b^ | 53 (54%) | 34 (49%) | 0.532 |  |
| Oxygen administration, n (%) ^b^ | 57 (58%) | 42 (60%) | 0.875 |  |
| In-hospital death, n (%) ^b^ | 11 (11%) | 10 (%) | 0.558 |  |
| Reason for ICU admission, n (%) |  |  |  |  |
| Circulatory disease ^b^ | 15 (15%) | 14 (20%) |  |  |
| Respiratory disease ^b^ | 8 (8%) | 7 (10%) |  |  |
| Abdominal/gastrointestinal disease ^b^ | 17 (17%) | 11 (16%) |  |  |
| Sepsis ^b^ | 31 (32%) | 17 (24%) |  |  |
| Renal/metabolic disease ^b^ | 13 (13%) | 11 (16%) |  |  |
| Trauma ^b^ | 9 (9%) | 6 (9%) |  |  |
| Other ^b^ | 5 (5%) | 4 (6%) |  |  |

^a^Values are shown as the mean ± SD.

^b^Values are shown as the number of patients (%).

^c^Values are shown as the median (interquartile range).

APACHE II: Acute Physiology and Chronic Health Disease Classification System II; BMI: body mass index; Hb: haemoglobin; ICU: intensive care unit

**Supplementary Table 3** Association between the magnitude of changes in vital signs and rSO_2_ at each time point in the mobilization process for survivors

|  |  | Rest –EHOB45° First 1 min | EHOB45° First 1 min –EHOB45° Last 1 min | EHOB45° Last 1 min –SEB First 1 min | SEB First 1 min –SEB Last 1 min | SEB Last 1 min –Supine |
| --- | --- | --- | --- | --- | --- | --- |
| HR | r | 0.025 | −0.004 | 0.229 | −0.108 | 0.061 |
| SBP | r | 0.305* | 0.126 | 0.223 | −0.162 | 0.189 |
| DBP | r | −0.037 | 0.407** | 0.041 | −0.155 | 0.186 |
| MAP | r | 0.080 | 0.299* | 0.137 | −0.184 | 0.168 |
| SpO_2_ | r | −0.078 | −0.098 | 0.118 | 0.111 | 0.109 |

*: *p* < 0.05, **: *p* < 0.01

DBP: diastolic blood pressure; EHOB: elevation of the head of the bed; HR: heart rate; MAP: mean arterial pressure; rSO_2_: regional cerebral oxygen saturation; SBP: systolic blood pressure; SEB: sitting on the edge of the bed; SpO_2_: percutaneous arterial oxygen saturation

**Supplementary Table 4** Association between the magnitude of changes in vital signs and rSO_2_ at each time point in the mobilization process for non-survivors

|  |  | Rest –EHOB45° First 1 min | EHOB45° First 1 min –EHOB45° Last 1 min | EHOB45° Last 1 min –SEB First 1 min | SEB First 1 min –SEB Last 1 min | SEB Last 1 min –Supine |
| --- | --- | --- | --- | --- | --- | --- |
| HR | r | −0.328 | 0.122 | −0.200 | 0.354 | −0.375 |
| SBP | r | −0.485 | 0.535 | 0.474 | 0.544 | 0.738* |
| DBP | r | 0.015 | −0.155 | 0.552 | 0 | −0.200 |
| MAP | r | −0.504 | 0.389 | 0.643* | 0.177 | 0.249 |
| SpO_2_ | r | 0.459 | −0.161 | 0.252 | −0.332 | 0.383 |

*: *p* < 0.05

DBP: diastolic blood pressure; EHOB: elevation of the head of the bed; HR: heart rate; MAP: mean arterial pressure; rSO_2_: regional cerebral oxygen saturation; SBP: systolic blood pressure; SEB: sitting on the edge of the bed; SpO_2_: percutaneous arterial oxygen saturation
